# Development and Validation of a Novel Gas-Washing Bottle Incubation System (GBIS) for Monitoring Microbial Growth in Liquid Media Under Well-Controlled Modified Atmosphere Conditions

**DOI:** 10.3390/foods13233723

**Published:** 2024-11-21

**Authors:** Seren Oguz, Eleonora Bonanni, Lotta Kuuliala, Mariem Somrani, Frank Devlieghere

**Affiliations:** 1 Research Unit Food Microbiology and Food Preservation (FMFP), Department of Food Technology, Safety and Health, Faculty of Bioscience Engineering, Ghent University, Coupure Links 653, B-9000 Ghent, Belgium; eleonora.bonanni@yahoo.it (E.B.); lotta.kuuliala@ugent.be (L.K.); mariem.somraniachouri@ugent.be (M.S.); frank.devlieghere@ugent.be (F.D.); 2 Research Unit Knowledge-based Systems (KERMIT), Department of Data Analysis and Mathematical Modelling, Faculty of Bioscience Engineering, Ghent University, Coupure Links 653, B-9000 Ghent, Belgium; 3 Departamento de Ingeniería Agronómica, Instituto de Biotecnología Vegetal, Universidad Politécnica de Cartagena, 30202 Cartagena, Spain

**Keywords:** constant modified atmospheres, gas flushing, sustainable packaging, *Listeria monocytogenes*

## Abstract

The transition towards more sustainable packaging calls for improving our ability to predict, control, and inhibit microbial growth. Despite the importance of modified atmosphere packaging (MAP) in food preservation, the exact relations between MAP gases (CO_2_, O_2_, N_2_) and microbial behavior are still poorly understood. Addressing this major knowledge gap requires a specific infrastructure to gain precise control over the gas composition during storage time. Thus, this study aimed at developing and validating an innovative gas-washing bottle incubation system (GBIS) with an adapted pH methodology for monitoring microbial growth in liquid media under different well-controlled conditions. *Listeria monocytogenes*—a psychrotrophic pathogen raising severe safety concerns under refrigerated conditions—was used as a representative microorganism. The results showed that daily gas flushing effectively dominated overnight headspace variations, allowing incubating *L. monocytogenes* and daily sampling for 13 days under static conditions. Subsequently, storage experiments were performed at 4 °C under stable pH and anaerobic conditions with different CO_2_ levels (20–40–60%). Significant growth reduction was observed from 6.0 to 4.8 log CFU/mL as CO_2_ increased from 20% (pH = 6.7) to 60% (pH = 6.2) (*p* ≤ 0.05). Overall, GBIS shows great potential in data collection for predictive modeling and, consecutively, for boosting decision-making in the food packaging sector.

## 1. Introduction

Due to the growing awareness of plastic pollution and demand for eco-friendly options, the food packaging industry is continuously striving for sustainable development. In recent years, numerous initiatives have focused on using recyclable or biodegradable materials instead of conventional and/or non-recyclable plastics to reduce packaging waste, reuse packaging materials, and save energy [1,2]. In the European Union (EU), the amended Directive 2018/852 [3] regarding packaging and packaging waste prioritizes the achievement of elevated levels of reusability and recyclability, with a goal to attain a 55% recycling rate of plastic packaging by 2030.

However, efforts to promote sustainable packaging solutions should go hand in hand with investigating their impact on microbial activity and subsequently on food safety. This becomes particularly evident when considering modified atmosphere packaging (MAP), a common preservation technology based on changing the headspace gas composition (typically consisting of CO_2_, O_2,_ and/or N_2_) to inhibit or limit microbial growth [4]. After closing the package, the atmosphere evolves throughout storage time as a result of several complex phenomena, including gas diffusion through the packaging materials, dissolving in the product, and microbial respiration [5]. This means that a given change, deviation, or defect in the packaging can have a profound impact on the atmosphere and thus microbial activity during storage time. For instance, when transitioning from conventional plastics to bio-based materials, choosing a more permeable film could lead to a faster loss of the protective atmosphere within the packaging. The fact that the desired atmosphere no longer exists may compromise the quality and/or safety of the packed product. Consequently, knowing that it would not be possible to test all possible packaging and storage scenarios one by one, there is a great need to improve our understanding of the relations between packaging configuration, atmosphere, and microbial growth [6,7].

It is crucial to consider microbial hazards in the context of sustainability as they impact food safety and significantly contribute to food loss. Particularly, *Listeria monocytogenes*—the cause of listeriosis with the highest mortality rate in the EU—poses a food safety risk in refrigerated MAP products due to its ability to grow at low temperatures [8,9,10]. To control the pathogens, especially *L. monocytogenes*, the combination of anaerobic conditions with high CO_2_ under refrigeration has generally been found to be the most effective MAP strategy [8,11,12,13,14,15]. In the field of predictive modeling, numerous studies have been carried out to collect quantitative data to assess how *L. monocytogenes* behaves under various MAP conditions [1,2,16,17]. However, most of these studies have been performed under naturally evolving MAP conditions, meaning that the exact relations between the individual gases and *L. monocytogenes* growth remain poorly understood.

Addressing the aforementioned knowledge gaps calls for novel approaches in data collection. Specifically, the exact relations between atmospheric gases and microbial growth can be examined by determining the growth parameters under constant (static) atmospheres. In the literature, there are applications of controlled atmospheres utilizing continuous gas circulation during the whole experiment that show great promise in the field of predictive microbiology [18,19,20,21]; however, these are still limited in number. Therefore, a specific tailored experimental setup is needed for monitoring microbial growth under well-defined and well-controlled conditions throughout storage time. Such a setup should allow precise control over the atmosphere as well as a fast equilibrium between the gas and the growth medium. While maintaining effective gas control, interconnected extrinsic factors—particularly CO_2_ dissolution and pH when using CO_2_-rich atmospheres [18,22]—should be considered, and the setup should allow individual monitoring of variables to understand their impact on microbial growth. Considering that effectively managing gas waste arising from continuous gas flow is crucial in scientific research due to its environmental and economic impacts [1], the setup should be both environmentally and economically friendly, avoiding constant gas flowing whenever possible. Also, the setup should facilitate easy sampling of the growth medium and upscaling, allowing the investigation of many intrinsic and extrinsic variables and their combinations. Moreover, to account for the need for extensive data collection, the setup should allow rapid and user-friendly sampling.

Hence, the present study aims to develop a gas-washing bottle incubation system (GBIS) for the investigation of the growth of microorganisms in liquid media under controlled modified atmosphere conditions. This study involved three types of experiments. Firstly, preliminary experiments were conducted to optimize technical and operational parameters, ensuring reliable and consistent results. Then, gas-controlling experiments were performed to observe the behavior of *L. monocytogenes* at 4 °C under CO_2_-rich gas mixtures while maintaining constant atmospheres throughout the experiment time (13 days). Finally, a pH monitoring method was developed and adapted to GBIS to carry out the pH experiments in the liquid medium, thereby monitoring pH under conditions with CO_2_ during the experiment.

## 2. Materials and Methods

### 2.1. Gas-Washing Bottle Incubation System (GBIS)

A gas-washing bottle incubation system (GBIS) was developed at the Research Unit Food Microbiology and Food Preservation (FMFP) at Ghent University. Throughout the development process, the following considerations were taken into account. First, the host growth medium for bacteria should be equilibrated at the desired gas composition. Secondly, the same headspace atmosphere conditions in several bottles connected in series should be maintained, enabling more data collection on microbial development under a given atmosphere. Next to that, each bottle in the setup should operate independently, offering a precaution against unforeseen issues that may arise in one of them. Lastly, sampling of both the growth medium and gas headspace should be possible without any interference with the atmospheric conditions. Thus, a commercially available gas-washing bottle (Euro-Scientific, Lint, Belgium) was modified by adding one outlet to its body.

**Figure 1:**
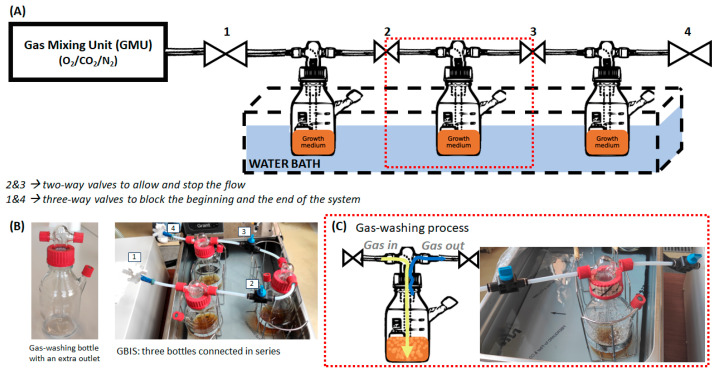
Schematic illustration of the gas-washing bottle incubation system (GBIS) with a water bath and gas mixing unit (GMU) (**A**), photographic illustration of one gas-washing bottle and GBIS immersed in the cooling water bath (**B**), and gas-washing process during flushing operation in the cooling water bath (**C**).

Figure 1A presents a schematic illustration of the GBIS with a gas mixing unit (GMU) and temperature control system. The GBIS consisted of three gas-washing bottles (500 mL) of glass connected in series by using polytetrafluoroethylene hoses (PTFE, Tameson, Eindhoven, the Netherlands) and valves (Tameson, Eindhoven, The Netherlands), as shown in Figure 1B. In this study, two-way valves (with the numbers 2 and 3) were used between each bottle to allow and stop the flow. These valves were chosen to ensure that the liquid did not flow back in successive bottles when gas flushing was stopped. Three-way valves (1 and 4) blocked the beginning and the end of the system when flushing was stopped, ensuring a closed system. A WITT gas mixer (MEM + Gas Mixers Series-WITT, Witten, Germany) and a cooling water bath with an immersion cooler unit (Grant Instruments, Cambridge, UK) were used as a GMU and temperature control system, respectively.

At the beginning of using the GBIS, the flowing gas composition (O_2_, CO_2,_ and N_2_) was set using the GMU. Headspace MAP gas analyzer CheckMate^®^9900 CO_2_/O_2_ (Dansensor A/S, Ringsted, Denmark) was used to check gas mixture composition expressed as the percentage of O_2_ and CO_2_. On the other hand, the changes in the headspace gas levels (∆CO_2_ and ∆O_2_) were expressed as percentage points. When the desired composition was achieved, the GMU was connected to the GBIS. All valves in the system were opened to allow for the gas to flow through the system (6.4 L/min); then, the gas mixture was introduced to the GBIS by flushing. During flushing, the gas flowed through the growth medium to saturate it with the desired gas, as demonstrated in Figure 1C. The gas-washing process begins with the gas entering the bottle and then being slowly blown into the vessel through a fritted glass tip (yellow arrow). After bubbling through the medium, the gas rises to the top and exits through the right-side tube (blue arrow). A green septum (Merck KGaA, Darmstadt, Germany) at an extra outlet enabled sampling of either gas or liquid for analyses. When flushing was decided to be stopped, firstly, valves 1 and 4 were closed simultaneously, and then valves 2 and 3 were closed also simultaneously. The last step was to separate the GBIS from the GMU.

### 2.2. Preliminary Experiments

As an indication of headspace gas/product volume ratio (G/P), the volume of a liquid medium (BHI: Brain heart infusion) in the gas-washing bottle was optimized by using 4 different volumes of BHI: 150 mL (3/1), 200 mL (3/2), 250 mL (1/1), and 300 mL (2/3) BHI for the flushing operation. Also, the volume of the medium is crucial to consider as the gas flowing through the medium caused bubbles on the surface to rise up, leading to possible flow from one bottle to another. After experiments, 200 mL (G/P:3/2) was found to be an optimum medium volume for the analyses in this study, as no issue with bubbling was observed.

The number of bottles in GBIS was optimized to three, as this number provided enough replicates for statistical analysis. Each bottle was employed to enable individual data collection on the behavior of the target microorganism (*L. monocytogenes* in this study) under the defined gas atmosphere. Additionally, this ensured compatibility with the size constraints of the cooling water bath used as a temperature control system to maintain the refrigerated conditions during the flushing operation.

When a defined gas mixture was provided, it gave rise to bubbling through the liquid medium in this setup; therefore, the liquid medium was expected to equilibrate with the headspace gas composition. For this, flushing duration was determined as the minimum time necessary to obtain a stable desired headspace gas atmosphere in the consecutive bottles. Three bottles were prepared with 200 mL BHI and connected. Then, the setup was connected to the gas mixing unit and exposed to an example MAP condition (CO_2_/O_2_/N_2_%) of 40/0/60% for 1 h. In addition, the headspace gas atmosphere inside bottles was checked every 5 min. It was observed that the gas atmosphere in the bottle became stable at the desired composition after 30 min of flushing.

The airtightness of the setup was checked by observing the changes in the gas headspace after the flushing operation was performed. The setup was first flushed with a defined gas mixture and then stored at 4 °C for 3 days. Overnight changes were checked for 1 day, 2 days, and 3 days of storage, respectively. This showed that the gas composition did not stay stable at the initial concentration. The change in gas composition was increasing as the storage time increased from 1 day to 3 days. Next to that, the frequency of the flushing operation was tested by performing flushing once a day and twice a day with an example gas composition (CO_2_/O_2_/N_2_%) of 40/0/60%. Overnight changes (∆CO_2_/∆O_2_) in the headspace gas composition, with the values of 0.9/0.03 and 0.9/0.025 percentage points the day after flushing once a day and twice a day, respectively, showed no difference in overnight gas variations between these two frequencies.

For this study, it was decided to perform flushing for 30 min and once a day to keep the atmosphere stable during the experiment. On the first day of flushing experiments, there was a need for pre-flushing before the inoculation process to replace the air atmosphere in liquid-medium-containing bottles with a desired modified atmosphere. Ten minutes of pre-flushing was found to be sufficient.

### 2.3. Bacterial Strain and Culture Conditions

*L. monocytogenes* strain ADQP105 isolated from smoked salmon was provided by ADRIA Développement (Quimper, France). The strain was stored as cryobeads in BHI broth supplemented with 15% glycerol (Chem-Lab, Zedelgem, Belgium) at −80 °C. Brain heart infusion (BHI, Oxoid, United Kingdom) was used as a growth medium in this study. Agar Listeria Ottaviani and Agosti (ALOA, Bio-Rad, Paris, France) was used as a selective medium for confirmation of *L. monocytogenes* while enumeration was performed on Tryptone Soya Agar (TSA, Oxoid, Basingstoke, UK) by spread plating. Serial dilutions were made in Peptone Physiological Salt Solution (PPS, Oxoid, Basingstoke, UK).

First, *L. monocytogenes* cryobeads were revived in 9 mL BHI and incubated overnight at 37 °C. Then, the BHI was streaked on ALOA plates and incubated at 37 °C for 48 h to check the strain purity. This is followed by one single colony being subcultured in a fresh 9 mL BHI, then incubated for 16 h at 37 °C. The second subculturing was carried out at 7 °C for 6 days in BHI broth to adapt the culture to low temperatures and ensure reaching the early stationary phase (approximately 9.5 log CFU/mL). Dilutions were made to reach an inoculum concentration of 10^3^ CFU/mL in 200 mL BHI-containing gas-washing bottles. The inoculum size was confirmed by plating on ALOA. Finally, prior to the first flushing operation, 1 mL of adequately diluted inoculum was introduced into the bottles by a syringe.

### 2.4. Gas-Controlling Experiments

Three gas-washing bottles with 200 mL of BHI broth were prepared and sterilized. To perform gas flushing experiments, these three bottles were connected consecutively in the biosafety cabinet to maintain a sterile working area. The GBIS was immersed in the cooling bath to guarantee that the temperature of the medium was maintained at refrigerated values during sampling and flushing. The flushing operation was performed once a day for 30 min during the 13-day experiment to keep headspace gas at the desired composition.

On the first day of flushing experiments, the liquid medium was sampled to confirm the initial concentrations of the medium in each bottle, and then the first flushing was performed. On the other days, the sampling of gas was followed by the sampling of the medium to determine the concentration of *L. monocytogenes* inside the bottles before each flushing. These samplings were repeated every day throughout the experiment.

Three MAP conditions were examined, as shown in Table 1, to demonstrate the stability of headspace gas during the experiment, allowing the evaluation of the impact of these gas mixtures on *L. monocytogenes* growth. Each condition was performed twice at two different times. Between flushing operations, the bottles were stored in a refrigerator at 3.9 ± 0.6 °C.

### 2.5. pH Experiments

The gas flushing experiments were performed in a similar manner to that described in Section 2.4. An anaerobic gas mixture of MAP4 (CO_2_/O_2_/N_2_%: 60/0/40) was performed at 7.1 ± 0.5 °C, with three experiments at three different times, to demonstrate pH evolution during the experiment and to determine the sampling intervals. Liquid samples were taken from bottles using a syringe, with a consistent sampling size of 1.5 mL. The pH measurements were conducted using a pH electrode (FC2020, Hanna Instruments, Temse, Belgium) connected to a pH meter (Hanna Edge HI2002, Hanna Instruments, Temse, Belgium).

**Figure 2:**
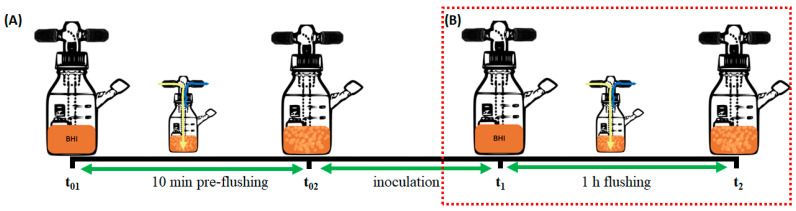
Time points for pH measurements are t_01_: before 10 min pre-flushing, t_02_: after 10 min pre-flushing/before inoculation, t_1_: after inoculation/before 1 h flushing, t_2_: after 1 h flushing on day 0 (**A**) and on days 4 and 8 (**B**).

Several processes were applied to a liquid medium throughout the experiment, requiring the assessment of their impact on the pH of the medium. Figure 2 illustrates sequential processes performed on the first day of the experiment, including 10 min pre-flushing, inoculation, and subsequent 1 h flushing, followed by daily 1 h flushing until the day before the last day. To determine whether a process made a difference in the pH of the medium or not, pH measurements were carried out on days 0, 4, 8, and 12 at different time points in a day in two experiments. Accordingly, pH measurement points were denoted as t_01_ (before 10 min pre-flushing), t_02_ (after 10 min pre-flushing/before inoculation), t_1_ (after inoculation/before 1 h flushing), and t_2_ (after 1 h flushing) on day 0 (Figure 2A), while the ones for days 4 and 8 were t_1_ and t_2_ (Figure 2B). The last pH measurement was conducted at the end of the experiment (day 12) as bottles were not subjected to flushing.

After the pH monitoring methodology was followed in two experiments, the liquid sampling size was optimized to 0.2 mL to observe pH changes more frequently under the same conditions. Thus, the third experiment was carried out by measuring the pH before each flushing operation every two days.

Based on the results of the pH analyses, the pH of the growth medium was determined under anaerobic conditions with three different CO_2_ levels (MAP1, MAP2, and MAP3) as detailed in Section 2.4. Hence, two individual 200 mL BHI-broth-filled bottles were employed to measure the initial pH of the growth medium after a 10 min pre-flushing process.

### 2.6. Statistical Analysis

Statistical analysis was conducted for microbial results at 4 °C. Daily cell counts—observed outcomes—obtained from bottles were converted to logarithmic values. Statistical analysis was performed with SPSS statistics 28. A three-way interaction general linear model with repeated measures was employed to examine the effects of multiple factors on the growth data. The model included two factors of interest, namely MAP conditions (MAP1, MAP2, and MAP3) and time points (0, 1, 2, 3, 4, 5, 6, 7, 8, 9, 10, 11, and 12). Also, the sequential position of the connected bottles (bottle 1, bottle 2, and bottle 3) corresponding to the order of gas flow in the system could be considered as another variable. This was taken as a covariate that may affect the results. Bonferroni correction was applied for multiple comparisons, while a level of significance of *p* ≤ 0.05 was chosen for all statistical analyses.

## 3. Results

### 3.1. CO_2_ and O_2_ Evolution

#### 3.1.1. Gas-Controlling Experiments

The gas mixtures initially introduced to GBIS remained at the desired levels throughout the 13-day-long experiment with a maximum deviation of 2.5 percentage points in CO_2_ concentration (%) and 0.10 percentage points in O_2_ concentration (%) observed under all studied atmospheres at 4 °C (Supplementary Table S1).

**Figure 3:**
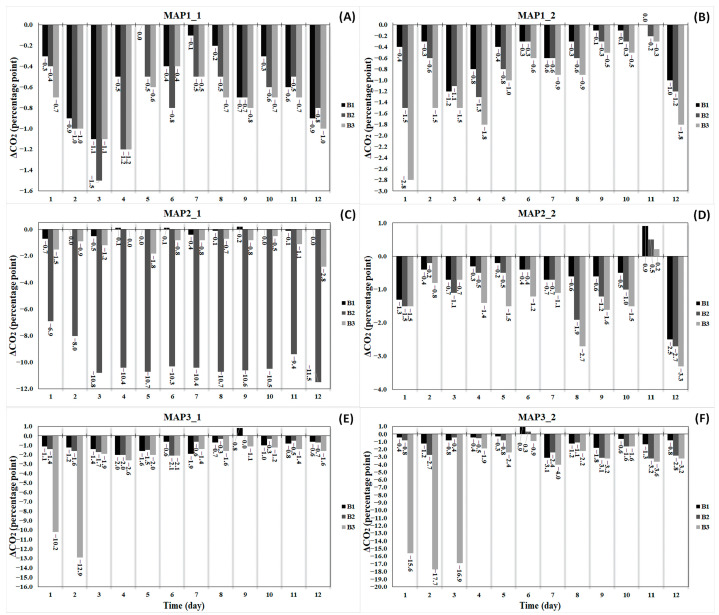
Overnight changes in headspace CO_2_ concentration in 1st bottle (B1), 2nd bottle (B2), and 3rd bottle (B3) under the following conditions CO_2_%/O_2_%/N_2_%: 20/0/80 for MAP1_1: 1st experiment (**A**) and MAP1_2: 2nd experiment (**B**), 40/0/60 for MAP2_1: 1st experiment (**C**) and MAP2_2: 2nd experiment (**D**), 60/0/40 for MAP3_1: 1st experiment (**E**) and MAP3_2: 2nd experiment (**F**).

Overnight decreases in CO_2_ concentration during 13 days in two experiments for each MAP condition are presented in Figure 3. Under all tested conditions at 4 °C, it was observed on some days that headspace CO_2_ concentration remained the same during the night. No more than a 1.5 percentage point reduction was seen in MAP1_1 (Figure 3A) during the experiment time. Similarly, MAP1_2 demonstrated minor decreases in CO_2_ levels (<3.0) (Figure 3B). Under the conditions of MAP1, slight differences in overnight CO_2_ concentration were seen between three consecutive bottles in each experiment. On the other hand, great differences between the bottles were noticed under the conditions of MAP2. As depicted in Figure 3C, the overnight drops in CO_2_ concentration ranged from 6.9 and 11.5 percentage points for B2 (second bottle) while they varied from 0 to 0.7 and 0 to 2.8 percentage points for B1 (first bottle) and B3 (third bottle), respectively. B2 was therefore considered a defective bottle owing to its unusual behavior of exhibiting a series of sharp decreases in CO_2_ concentration throughout the experimental duration, thereby distinguishing it from the remaining two interconnected bottles. In MAP2_2, all bottles have minor changes of up to 3.3 percentage points (Figure 3D). Under MAP3, only B3 in each experiment showed a reduction of over 10 percentage points at the beginning of the experiments that was not observed in the rest of the experiments. All the bottles in MAP3_1 showed similar changes in CO_2_ levels of up to 2.1 percentage points (Figure 3E), whereas the bottles in MAP3_2 showed changes of up to 4 percentage points (Figure 3F).

**Figure 4:**
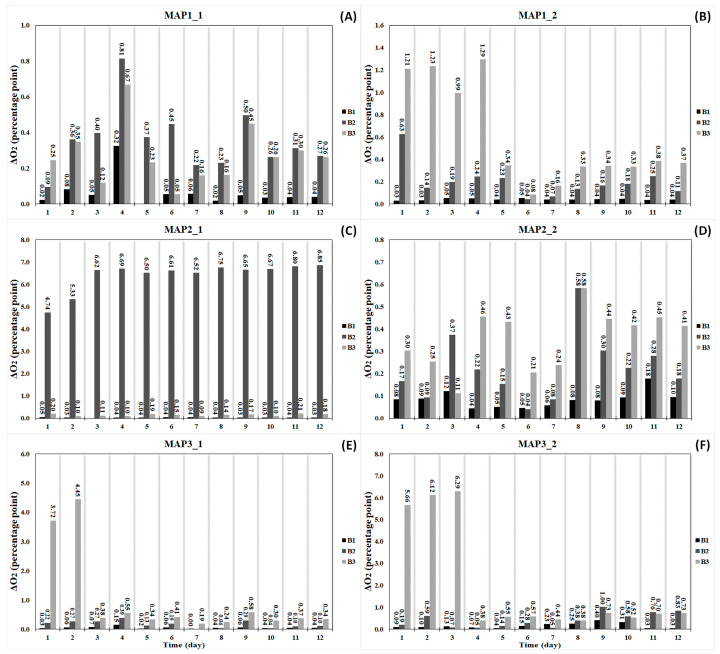
Overnight changes in headspace O_2_ concentration in 1st bottle (B1), 2nd bottle (B2), and 3rd bottle (B3) under the following conditions CO_2_%/O_2_%/N_2_%: 20/0/80 for MAP1_1: 1st experiment (**A**) and MAP1_2: 2nd experiment (**B**), 40/0/60 for MAP2_1: 1st experiment (**C**) and MAP2_2: 2nd experiment (**D**), 60/0/40 for MAP3_1: 1st experiment (**E**) and MAP3_2: 2nd experiment (**F**).

The same bottles where headspace CO_2_ content decreased also showed an increase in O_2_ concentration (Figure 4). While the consecutive bottles demonstrated slightly different increases as seen in each experiment, some exceptions were realized. The highest increments (>1 percentage point) in headspace O_2_ concentration made by B3 at the beginning of the experiment were not seen within time (Figure 4B,E,F). However, the defective bottle—B2—also exhibited abrupt increases in O_2_ concentration levels over the course of the experimental duration as depicted in Figure 4C. When the abovementioned bottles, which demonstrated noticeable increments, were not considered, the overnight increments in O_2_ seen in bottles ranged between 0 and 1 percentage points.

#### 3.1.2. pH Experiments

Regarding the condition of MAP4 (CO_2_/O_2_/N_2_:60/0/40) at 7 °C, studied to observe pH evolution during the experiment, overnight changes in headspace gas concentration during 13 days in nine bottles were analyzed (Supplementary Figure S1). Small changes of up to 4 percentage points and up to 0.7 percentage points were observed in CO_2_ and O_2_ concentration, respectively. However, one bottle (the first bottle in the third experiment) exhibited a sharp decrease of 18.7 percentage points in CO_2_ content and an increase of 7.6 in O_2_ content near the end of the experiment. These changes were not observed in the rest of the experiment.

### 3.2. pH Monitoring Methodology: The Impact of Pre-Flushing, Inoculation, and Flushing Processes on the pH of the Medium

**Figure 5:**
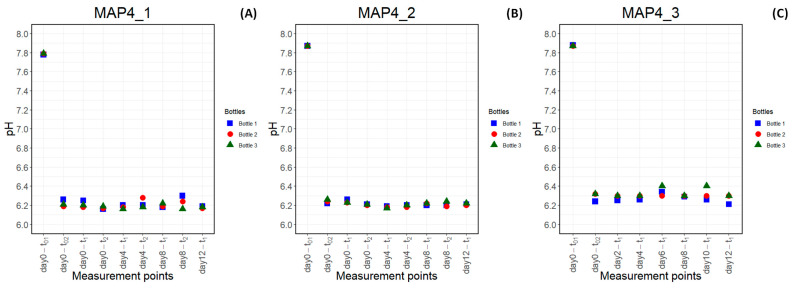
Change in the pH of BHI medium at 7 °C under the following condition (CO_2_%/O_2_%/N_2_%): 60/0/40 for MAP4_1:1st experiment (**A**), MAP4_2: 2nd experiment (**B**), and MAP4_3: 3rd experiment (**C**) over time points t_01_: before 10 min pre-flushing, t_02_: after pre-flushing/before inoculation, t_1_: after inoculation (only on day 0)/before 1 h flushing, t_2_: after 1 h flushing.

Figure 5A,B show the evolution of pH in three individual bottles across days 0, 4, 8, and 12 in two experiments of MAP4 (CO_2_/O_2_/N_2_%: 60/0/40). The pH of BHI after sterilization was recorded as 7.79 ± 0.01 and 7.87 ± 0.00 in the first and second experiments, respectively. It was observed that the first introduction of the gas atmosphere (10 min pre-flushing) dramatically altered the pH of the medium to 6.22 ± 0.04 in the first experiment and to 6.24 ± 0.02 in the second experiment. Then, the pH of the medium stayed at the value recorded after pre-flushing/before inoculation on day 0 (day 0-t_02_) without any further acidification throughout the experiment, meaning that the inoculation and 1 h flushing processes did not change the pH. Thus, pH was monitored based on pH measurements taken before each flushing operation (t_1_), as shown in the third experiment (Figure 5C). As in the other two experiments, the 10 min pre-flushing on day 0 changed pH from 7.87 ± 0.01 to 6.29 ± 0.05, which remained stable for the rest of the experiment. Therefore, the pH of the growth medium was found to be 6.23 ± 0.05 (average) during the experiment under this condition.

Based on the findings shown in Figure 5, the pH of the three MAP conditions studied in Section 2.4 was determined by pH measurement taken after 10 min pre-flushing regardless of whether the inoculation process was performed or not. The pH of the medium after 10 min pre-flushing was found to be 6.73 ± 0.04, 6.34 ± 0.03, and 6.21 ± 0.02 under the gas compositions (CO_2_/O_2_/N_2_%) of 20/0/80, 40/0/60, and 60/0/40, respectively.

### 3.3. The Effect of High CO_2_ and Low O_2_ on the Growth of Listeria Monocytogenes

#### 3.3.1. Gas-Controlling Experiments

Daily microbial counts from each bottle were collected throughout the experiment time and then used to obtain individual growth curves for each experiment under three different MAP conditions (Supplementary Figure S2). Although overnight fluctuations in CO_2_ and O_2_ levels were observed in varying amounts among the bottles under a defined atmosphere, similar growth curves were exhibited in all bottles under each condition for 13 days, except for the growth curve in B2 (second bottle) in MAP2_1 (CO_2_%/O_2_%/N_2_%: 40/0/60). For instance, three growth curves under MAP1_2 (CO_2_%/O_2_%/N_2_%: 20/0/80) in Figure S2B were closely aligned while B3 (third bottle) showed higher variation in headspace gases at the beginning of the experiment compared to other bottles (Figure 3B and Figure 4B). Additionally, under the same gas atmospheres, the growth curves obtained in the first and the second experiments were similar (Figure S2A,B). Therefore, the small overnight gas fluctuations did not seem to influence the growth curves under all defined atmospheres, indicating that the microbial growth remained consistent with the standard deviations ranging from 0 to 0.3 log CFU/mL despite these variations. However, B2 in MAP2_1 was considered defective when headspace gas evolution in bottles was analyzed (in Section 3.1.1, Figure 3C and Figure 4C). In addition, it was observed that the difference between *L. monocytogenes* growth data obtained in B1 (first bottle) and B2 was becoming more noticeable as the end of the experiment approached (Figure S2C). These major variations (approximately 0.5–0.6 log CFU/mL) realized between these consecutive bottles of the GBIS for MAP2_1 were not encountered in the other experiments. Hence, this defective bottle was discarded from the statistical data analyses.

**Figure 6:**
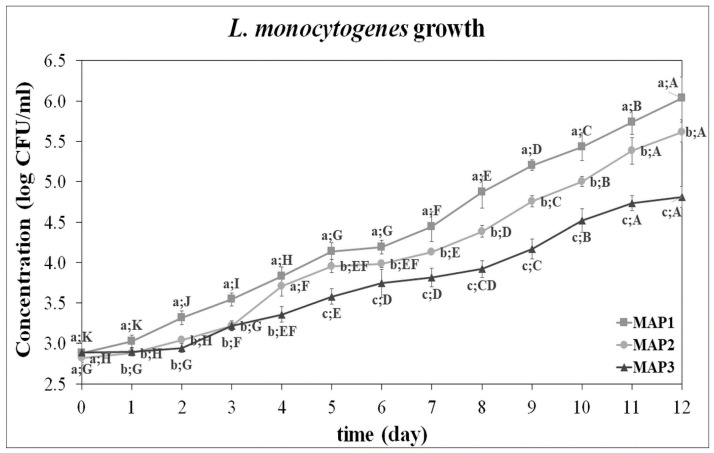
Growth curves of *L. monocytogenes* within a 13-day experiment for the following conditions CO_2_%/O_2_%/N_2_%: 20/0/80 (MAP1), 40/0/60 (MAP2), 60/0/40 (MAP3). Labels with different uppercase and lowercase letters are significantly different (*p* ≤ 0.05) among time points and tested atmospheres, respectively (error bars denote standard deviation, *n* = 6 for MAP1 and MAP3 and *n* = 5 for MAP2).

Figure 6 summarizes the growth of *L. monocytogenes* under the three investigated atmospheres and shows whether there is a significant difference between time points and MAP conditions or not. It was found that microbial concentrations under MAP1 (pH = 6.7), MAP2 (pH = 6.3), and MAP3 (pH = 6.2) significantly increased in 13 days from 3.0 ± 0.1 to 6.0 ± 0.3, 5.6 ± 0.1, and 4.8 ± 0.1 log CFU/mL, respectively. A significant reduction in microbial growth was observed as CO_2_ concentration increased (*p* ≤ 0.05). As can be seen in the graph, while the stationary phase was not reached within 13 days, the growth curves exhibit the durations of lag time, which are approximately 1 day, 2 days, and 2 days for MAP1, MAP2, and MAP3, respectively.

#### 3.3.2. pH Experiments

The growth curves in three experiments under the condition of MAP4 (CO_2_/O_2_/N_2_%: 60/0/40) were closely aligned (Supplementary Figure S3). Also, microbial concentrations under this condition (pH = 6.23 ± 0.05) increased in 13 days from 2.9 ± 0.1 to 7.4 ± 0.2 log CFU/mL. When compared to the same gas mixture at 4 °C (MAP3), the concentration of *L. monocytogenes* at the end of the experiment (pH = 6.21 ± 0.02) was 2.6 log CFU/mL higher at 7 °C.

## 4. Discussion

The GBIS has a good gas-controlling capacity, as overnight changes in headspace CO_2_ and O_2_ concentration were controlled by flushing on a daily basis. These changes occurring during the night were seen as the setup was not completely airtight. In preliminary experiments (Section 2.2.), once a day 30 min flushing was found to be enough to reach equilibrium between the liquid medium and a defined gas atmosphere as well as to compensate for the overnight fluctuations, which had no impact on the growth patterns. Applying daily flushing is a very important advantage of the GBIS for investigating the effect of a constant gas mixture on the microorganism growth in a liquid medium.

When daily information on headspace gas composition during the experiment was examined, it was possible to easily detect any bottle with unusual behavior (as mentioned in Section 3.1.1). In this study, major fluctuations in headspace gas composition were exhibited by only one bottle within the system (Figure 3 and Figure 4). Sharp changes, observed in some bottles only at the beginning of the experiment, were quickly compensated over time by adjusting connection elements to enhance the airtightness of the GBIS in addition to daily flushing. Nonetheless, large deviations were still observed in one bottle throughout the experiment (Figure 3C and Figure 4C). While major changes were seen in only one bottle in the setup, the remaining bottles in the same setup did not show these changes. It is noteworthy that the presence of only one atypical bottle in the interconnected system did not have any adverse impact on the gas-controlling capacity of the other two bottles. Therefore, this observation provided valuable insight into the concept that each bottle was functioning independently by utilizing the GBIS.

Data collection on the growth of microorganisms provides insight into how they are linked to environmental factors [23,24]. When these factors are not gas-related such as pH, water activity, concentration of acids, and temperature, automation methods facilitate this data collection [17]. However, the complexity of food matrices poses challenges, particularly in discerning the precise relationships between atmospheric gases (CO_2_, O_2_, N_2_) and microbial growth. Growth experiments in liquid and/or solid food exposed to a specific gas atmosphere during the entire experiment are more complex as many factors influence the gas concentration during the experiment. Consequently, the fundamental interactions between different MAP gases and microbial growth remain poorly understood. Achieving this goal requires gaining precise control over growth conditions as a crucial initial step. The present study showed that the GBIS is a useful tool for data collection to investigate the behavior of microorganisms in a liquid medium depending on the well-defined gas headspace composition. Using a standardized medium (in this study, BHI broth) provided a controlled and consistent environment. Achieving such control would be challenging in food items due to the unpredictable interactions of intrinsic factors (e.g., food chemical composition) and implicit factors (e.g., background flora) with the target culture. Also, the analysis of the daily data collection enabled the identification of a defective bottle, which was subsequently excluded from the evaluation (as mentioned in Section 3.3.1). Hence, this study provides reliable estimations of microbial growth in a liquid medium exposed to specific gas atmospheres. This can easily be expanded to the investigation of different variable combinations in liquid environments within this single setup, thereby streamlining data collection and analysis, and contributing valuable data for the establishment of predictive microbiology models.

The development of the GBIS fulfilled the long-standing desire to conduct experiments under static gas conditions for the development of predictive models. In MAP systems, it is well known that dynamic O_2_ and CO_2_ gradients exist between the headspace of the package and the surroundings, as well as with the food throughout storage time. It was previously stated by Devlieghere et al. [25] that CO_2_ is partially dissolved in the water phase of the product when included within the packaging. It is unique and special in its high solubility in an aqueous solution. High dissolution causes a change in headspace gas composition or partial pressure from the initial state of inclusion in the food package [8] which was also observed by Couvert et al. [18]. On the other hand, Hurme [26] reported that not only CO_2_ dissolution into the product but also leakage in the package caused a drastic decrease in headspace composition after the initial gas flushing. The final concentration could be lower than half the original one. Also, regarding the anaerobic packaging, a trace level of O_2_ remains in the package because of inefficient flushing and will affect the behavior of the microorganism contaminating the food product [21,27,28]. Guillard et al. [16] conducted research on predicting the growth of the same *L. monocytogenes* strain as used in the present study, under dynamic gas changes, and carried out the analysis of gas exchange in the packaging system (tray + lid) composed of a material used that was very permeable. They indeed observed that CO_2_ and O_2_ contents quickly decreased and increased, respectively. According to these authors, despite the inclusion of high CO_2_ levels at the moment of packaging, this quick dissipation of CO_2_ within the packaging headspace resulted in a very limited observed effect of CO_2_ on the growth of the same *L. monocytogenes* strain in inoculated foods. Moreover, changes in gas composition during storage may result from the dissolution of CO_2_ and O_2_ as well as from gas production/consumption due to microbial reactions [18,29]. Therefore, although the desired gas atmosphere is initially flushed into the packages, it will no longer be at the same composition causing unreliable estimations of target microorganisms’ growth.

A few researchers attempted to control the headspace gas atmosphere together with other parameters, namely pH and temperature, during their growth experiments [18,19,20,21,30]. Bennik et al. [30] previously highlighted the need for careful control of atmospheric conditions to inhibit pathogen growth on agar surfaces while investigating *Listeria* growth under the effect of varying O_2_ and CO_2_ using a solid model system. The model system enabled continuous gas flow so that CO_2_ concentrations would not be influenced by CO_2_ dissolution. In the setup, flasks containing buffered agar plates were connected with silicone tubing and incubated in a climatized room. Similarly, Hendricks and Hotchkiss [19] conducted a study on the effect of a constant CO_2_ content on *L. monocytogenes* growth in a buffered nutrient broth where pH and atmosphere conditions were kept constant. In their study, a gas-tight chamber inside a low-temperature incubator was continuously flushed with the desired gas mixture to make microbial respiration in culture plates ineffective on gas composition. Unlike this system, the GBIS allowed for bubbling within the inoculated liquid medium in a gas-washing bottle, thereby facilitating gas circulation within it while maintaining a stable headspace gas composition. In other words, the entire medium was treated with the desired gas composition. In another study, Couvert et al. [18] worked on monitoring the growth of the same *L. monocytogenes* strain as in the current study, but in buffered BHI containing glucose and yeast extract, in static conditions of atmospheric carbon dioxide, temperature, and pH. Their protocol indicated the use of a bubbler system with flasks to control headspace CO_2_ atmospheric composition with a continuous gas circulation during the entire growth curve acquisition so that CO_2_ dissolution would not affect partial pressures in the gas atmosphere. In the field of scientific research, minimizing gas waste is of utmost significance due to its environmental and economic implications [1]. With the GBIS, daily 30 min flushing was applied to a liquid medium in gas-washing bottles to keep the desired gas headspace constant rather than continuous gas flowing. Couvert et al. [21] used a hypoxic chamber where the same *L. monocytogenes* strain inoculated BHI agar plates incubated during the experiment to set O_2_ concentration to the desired composition and keep it constant during the experiment. By using the GBIS, growth data under well-controlled CO_2_ and O_2_ concentrations were collected from daily replicate experiments, which were carried out in a liquid growth medium in three bottles during the entire experimental time.

CO_2_, in liquid media, is known to lower the pH of the medium through a specific reaction depending on factors such as pH, temperature, and gas content. This reaction starts with CO_2_ reacting to form carbonic acid (H_2_CO_3_), which itself reacts to form carbonate ions (CO_3_^2−^) and bicarbonate ions (HCO_3_^−^). The release of hydrogen ions (H⁺) decreases the pH. Thus, it might be unclear whether the reducing effect on microbial growth is a result of high CO_2_ concentration or lowered pH [18,22]. Most studies relating to the CO_2_ effect on this pathogen have considered its role in acidifying the medium [16,18,19]. Based on this need, GBIS was further developed to collect data on pH through the adaptation of a reliable pH monitoring method. This method allowed precise control over the pH of the growth medium while desired gas atmospheres were kept stable. Therefore, the pH of a standardized BHI medium was monitored without any addition, differing from the studies in which the pH was partially controlled or controlled by utilizing a buffered liquid growth medium to minimize medium acidification caused by CO_2_ dissolving in the medium [18,19,20,30].

According to Lekesiz and Oflaç [1], the sustainable packaging practices model (SPPM) has primarily focused on practices such as sustainable raw material sources and processes, recyclability, reducing packaging material and carbon footprint, and saving energy. It mainly focused on reducing the environmental impact of the packaging itself by reusing or recycling packaging materials. In the transition to more environmentally friendly options, it is crucially important to understand the exact relations between extrinsic parameters and microorganism growth. Given that *L. monocytogenes* is a critical food safety concern in sustainable packaging, the current study not only presented a setup allowing for monitoring the microorganism growth under static conditions but also addressed the growth of this foodborne pathogen in practice to validate the effectiveness of the GBIS. Our study also aims, in the long term, to improve sustainable packaging design by providing reliable and extensive data for the prediction of *L. monocytogenes* behavior under different well-controlled gas atmospheres. These data can be used to estimate the effect of using an alternative, more sustainable packaging concept on the microbial safety of the packaged product, combining estimations regarding sustainability as well as microbial safety.

This study showed the effect of a well-defined high CO_2_ and low O_2_ concentration on *L. monocytogenes* growth at 4 °C (Figure 6). Generally, for maximum effectiveness and benefits of MAP, different gases individually or in combination are selected based on their properties [31]. Among pathogenic bacteria, *L. monocytogenes* is less affected by gas atmospheres compared to Gram-negative bacteria and strict aerobic and anaerobic pathogens [8,21]. To be able to reduce and even inhibit the growth of *L. monocytogenes*, the benefit of the use of MAP relies on the increase in CO_2_ and the reduction in O_2_ levels combined with low temperatures [18]. According to Gokoglu [11], this combination inhibits psychrotrophic microorganisms in seafood. Yilmaz et al. [15] investigated the growth of *L. monocytogenes* in rainbow trout fillets stored at 4 °C under MAP (CO_2_:O_2_%) with 50:0, 20:80, and 90:2.5 and other packaging environments: air and vacuum. They found that packing with MAP resulted in the slowest growth of *L. monocytogenes*. In another study conducted by Rutherford et al. [13], RTE shrimps were inoculated with a cocktail of five different strains of *L. monocytogenes*, and growth was investigated in different packaging environments (air, vacuum, and MAP with 100% CO_2_) and at different temperatures (3, 7, and 12 °C) for 15 days. This study demonstrated that storing in MAP with high CO_2_ and at 3 °C did not allow the growth of *L. monocytogenes* throughout the storage time, whereas other combinations of packaging conditions and temperature did. Also, Hauzokim et al. [14] reported that *L. monocytogenes* growth in smoked salmon was reduced due to the inhibitory effect of CO_2_ when low temperature was maintained during anaerobic MAP. Overall, these studies showed that high CO_2_ exerts its inhibitory effect when combined with refrigerated temperatures, with the effect becoming more pronounced as the temperature decreases. The current study supports the reduced effectiveness of CO_2_ by observing the growth of *L. monocytogenes* under CO_2_-enriched conditions at 7 °C (MAP4), though further investigation is needed to strengthen this hypothesis.

Depending on the type of product, one of the important goals of atmosphere modification is to generate sufficiently low O_2_ conditions, which can improve food preservation by reducing metabolic processes, respiration rate, oxidative stress, and negative effects on quality, such as color change and rancidity [21,27,28]. Hendricks and Hotchkiss [19] investigated *L. monocytogenes*, in buffered nutrient broth at 7.5 °C, in modified atmospheres (30% CO_2_) with four different O_2_ concentrations (0, 10, 20, and 40%). They found that the O_2_-free atmosphere was the one where *L. monocytogenes* growth slowed down the most. On the other hand, in the study conducted by Bennik et al. [30], the increase in O_2_ level from 1.5% to 21% did not have any impact on the growth of *L. monocytogenes*. Similarly, Couvert et al. [21] observed that growth rates of the same *L. monocytogenes* strain as in the current study were not affected by the increases in O_2_ levels from 0.1% to 0.5% and from 0.1% to 21%. This finding is in line with our findings that the effects of the slight overnight O_2_ increase seen in the bottles on *L. monocytogenes* growth were negligible.

While *L. monocytogenes* was able to significantly grow at 4 °C for 13 days, increasing CO_2_ content significantly reduced the growth (*p* ≤ 0.05). Similarly, in a study carried out by Provincial et al. [12], *L. monocytogenes* growth in sea bream at 4 °C was analyzed under different modified atmospheres (CO_2_/O_2_/N_2_%: 60/0/40, 70/0/30, and 80/0/20). They found that increasing CO_2_ slowed the growth significantly. Analogous results were obtained by Farber et al. [32] while investigating five *L. monocytogenes* strains in BHI. They also concluded that the major effects of CO_2_ on *L. monocytogenes* are bacteriostatic, decreasing growth rate and increasing lag time, and these effects were seen when packed in MAP with CO_2_ concentration above 50%. This finding could explain the difference between MAP conditions seen during the first 4 days of the experiment. The significant differences were mostly observed between growth curves under MAP1 (pH = 6.7) and MAP3 (pH = 6.2) (Figure 6). Furthermore, it was found to be consistent with the characteristics summarized by Floros and Matsos [28] that the bacteriostatic effect of CO_2_ depends on its concentration and the temperature at which the food is stored. CO_2_ slows down microbial growth when present at concentrations ≥ 20%–30%, depending on the product. The bacteriostatic effect of CO_2_ can be explained briefly in four activity mechanisms. The first one is the changing of the cell membrane function, which enables taking up nutrients and absorption. Secondly, it inhibits the enzymes or decreases the rate of enzyme reactions. The third one is the penetration of bacterial membranes, resulting in intracellular pH changes. Lastly, it causes direct alterations in the physicochemical properties of proteins [33]. While the indirect effect of high CO_2_ concentration on the microorganism growth is through changing the pH of the growth medium, some researchers concluded that the observed inhibition is likely a result of controlled CO_2_ itself [18,19,30]. Also, no significant relationship was found between the pH changes during the experiment and the growth of *L. monocytogenes* [34]. For the same *L. monocytogenes* strain as in the current study, Couvert et al. [18] observed a similar inhibitory effect of high CO_2_ concentrations at both pH values of 5.5 and 7.0.

The initial pH and final pH value of the medium depend on the amount of CO_2_ and the final maximum concentration of *L. monocytogenes*, respectively, both of which contribute to the acidification of the medium [21,30]. In this study, the stable pH over time indicated the initial acidification of the medium was due to dissolving CO_2,_ and the equilibrium between the gas composition and liquid medium was achieved (Section 3.2). No further acidification was observed from the growth of *L. monocytogenes* when the maximum concentration reached 7.4 log CFU/mL at 7 °C. Therefore, pH was expected to remain constant at 4 °C, where the final concentrations were lower (Section 3.3.1) than the one at 7 °C.

The experimental setup as a whole used in the current study is adaptable, allowing for the inclusion of additional bottles and the use of alternative systems, such as gas mixing units and temperature control (e.g., cold room), depending on specific experimental requirements. Monitoring the growth of microorganisms has been a crucial aspect of other food studies, such as fermentation studies, leading researchers to develop methods to gain extensive information. Similar to the current study, Anderlei and Büchs [35] identified shortcomings in previous studies and introduced a novel method for the online measurement of the oxygen transfer rate in shaking flasks. They conducted parallel experiments and gained valuable insights into culture and fermentation conditions, encouraging them to further improve their setup. They created a respiration activity monitoring system (RAMOS) that enabled the measurement of carbon dioxide transfer rate and respiratory quotient in shaking bioreactors [36]. Moreover, researchers highlighted the importance of precise CO_2_ monitoring in cell cultures, as it significantly impacts cellular metabolism, product quality, and pH regulation in culture media. However, traditional methods are invasive and unsuitable for small-scale cultures, lacking appropriate monitoring systems. This called for noninvasive techniques to improve accuracy and reliability without any direct contact with sensors or contamination risks with the culture medium [37,38]. Therefore, the GBIS, introduced in the present study with such advantages, not only could be found applicable for monitoring microorganisms under controlled conditions but also holds promise for fermentation and cell culture studies with further developments.

The present study has some limitations. Firstly, the GBIS was designed for studying microbial developments in liquid growth media, limiting its application to liquid environments rather than solid environments. Secondly, airborne microorganisms cannot be studied using this system. Thirdly, no criteria such as the relative standard deviation could be determined for assessing the consistency of overnight headspace gas variations. In this study, overnight fluctuations in headspace gas levels did not influence the behavior of the *L. monocytogenes* at 4 °C under three anaerobic conditions with different CO_2_ levels. Based on the complete data analysis, including headspace gas evolution and growth data, the acceptable variation limit changes depending on the specific strain, temperature, and tested conditions. Consequently, any study utilizing a GBIS should report the full dataset, and the effect of gas variations on growth should be evaluated in the context of the specific strain and experimental conditions. Lastly, the focus of the current study was on controlling the gas headspace composition and temperature throughout the experiment, thereby obtaining daily and reliable growth data by utilizing the novel setup GBIS. Therefore, the findings on *L. monocytogenes* growth under well-defined gas atmospheres and refrigerated temperatures can be leveraged in further investigations. By enhancing the GBIS, deeper insights into the growth of this pathogen can be gained, particularly by studying varying extrinsic factors, such as O_2_, temperature, and the pH of the medium.

## 5. Conclusions

In this study, a novel setup, the GBIS, was first developed, and its effectiveness was validated by investigating *L. monocytogenes* growth at 4 °C under anaerobic modified atmospheres with elevated CO_2_ levels of 20%, 40%, and 60% for 13 days. Using the GBIS, the growth was successfully monitored, and the sampling was performed daily. Although there were slight changes observed in headspace gas composition in the bottles overnight, flushing once a day was successful in compensating for these changes, ensuring that they had no impact on the growth pattern. With gas controlling capacity, gas atmospheres were kept at the desired level by using the GBIS. *L. monocytogenes* growth was significantly reduced by the increase in CO_2_ content when stored in the combination of refrigeration with anaerobic MAP conditions. The pH of the medium was stable under these CO_2_-rich gas mixtures throughout the experiment.

The current study was the first and essential step in the transition towards sustainable packaging. The GBIS was found to be an innovative and effective tool for obtaining reliable growth data of foodborne pathogens in a liquid medium under static conditions of different gas atmospheres. This data collection may have important implications for food safety and preservation, as it highlights the importance of selecting the appropriate MAP condition to control microbial growth and reduce food waste by extending the products’ shelf life. The prediction of *L. monocytogenes* growth and survival—under not only various MAP conditions but also other parameters—is needed for assessing the safety and shelf life of food products. Understanding the effect(s) of pH, temperature, and CO_2_ requires a comprehensive study where the individual effects can be examined, addressing a broader and more detailed scope. Thus, the GBIS can be enhanced to allow subsequent large-scale data collection on how parameters, such as aerobic atmospheres, temperature, dissolved gases, and pH, affect *L. monocytogenes* growth to promote sustainable packaging by adding non-destructive tools or sensors as well as the addition of more individual bottles. It may also be further developed for the investigation of microorganism growth under the effect of the dynamic exchange of gases surrounding the food matrices. The insights gained from this innovative approach could be set to benefit biotechnological studies such as cell cultures and fermentation studies by offering robust methodologies, especially for studying controlled gas atmospheres.

## Figures and Tables

**Table 1:** Modified atmosphere packaging (MAP) conditions examined by using the gas-washing bottle incubation system (GBIS).

Conditions	T (°C)	Gas Mixture (CO_2_/O_2_/N_2_%)		
		O_2_ (%)	CO_2_ (%)	N_2_ (%)
**MAP1** MAP1_1 (1st experiment) MAP1_2 (2nd experiment)	4	0	20	80
**MAP2** MAP2_1 (1st experiment) MAP2_2 (2nd experiment)	4	0	40	60
**MAP3** MAP3_1 (1st experiment) MAP3_2 (2nd experiment)	4	0	60	40

## Data Availability

The original contributions presented in this study are included in the article/Supplementary Materials. Further inquiries can be directed to the corresponding author.

## Supplementary Materials

The following supporting information can be downloaded at https://www.mdpi.com/article/10.3390/foods13233723/s1: Table S1: Minimum and maximum levels (%) of O_2_ and CO_2_ in the measured gas mixtures introduced to the gas-washing bottle incubation system (GBIS) for each condition during 13 consecutive days; Figure S1: Overnight changes in headspace CO_2_ and O_2_ concentration in 1st bottle (B1), 2nd bottle (B2), and 3rd bottle (B3) under the following condition CO_2_%/O_2_%/N_2_%: 60/0/40 at 7 °C for MAP4_1: 1st experiment (A and D), MAP4_2: 2nd experiment (B and E), and MAP4_3: 3rd experiment (C and F). Figure S2: Growth curves obtained from 1st bottle (B1), 2nd bottle (B2), and 3rd bottle (B3) for following conditions CO_2_%/O_2_%/N_2_%: 20/0/80 for MAP1_1: 1st experiment (A) and for MAP1_2: 2nd experiment (B), 40/0/60 for MAP2_1: 1st experiment (C) and MAP2_2: 2nd experiment (D), 60/0/40 for MAP3_1: 1st experiment (E) and MAP3_2: 2nd experiment (F). Figure S3: Growth curves of *L. monocytogenes* within a 13-day experiment under the following condition CO_2_%/O_2_%/N_2_%: 60/0/40 for MAP4_1: 1st experiment, MAP4_2: 2nd experiment, and MAP4_3: 3rd experiment (error bars denote standard deviation, *n*=3 for MAP4_1 MAP4_2 and MAP4_3).
