# Supplementary material for: Development and Validation of a Novel Gas-Washing Bottle Incubation System (GBIS) for Monitoring Microbial Growth in Liquid Media Under Well-Controlled Modified Atmosphere Conditions
Source: Foods. 2024 Nov 21;13(23):3723. doi: 10.3390/foods13233723 (PMC11640465; doi:10.3390/foods13233723)
Supplement: Supplementary file 1 [file foods-13-03723-s001.zip › foods-3318053-supplementary.pdf]

Supplementary Material

Table S1. Minimum and maximum levels (%) of O_2 and CO_2 in the measured gas mixtures introduced to the gas-washing bottle incubation system (GBIS) for each condition during 13 consecutive days

**Conditions (O_2%/ CO_2%/ N_2%)**	**Flushing gas composition**	
	**O_2% (min:max)**	**CO_2% (min:max)**
**MAP1 (0/20/80)**		
MAP1_1 (1st experiment)	0.01 : 0.09	20.1 : 22.4.
MAP1_2 (2nd experiment)	0.00 : 0.01	20.0 : 21.1
**MAP2 (0/40/60)**		
MAP2_1 (1st experiment)	0.00 : 0.01	40.0 : 41.0
MAP2_2 (2nd experiment)	0.00 : 0.01	40.0 : 41.7
**MAP3 (0/60/40)**		
MAP3_1 (1st experiment)	0.01 : 0.10	60.0 : 61.6
MAP3_2 (2nd experiment)	0.01 : 0.02	60.0 : 62.5

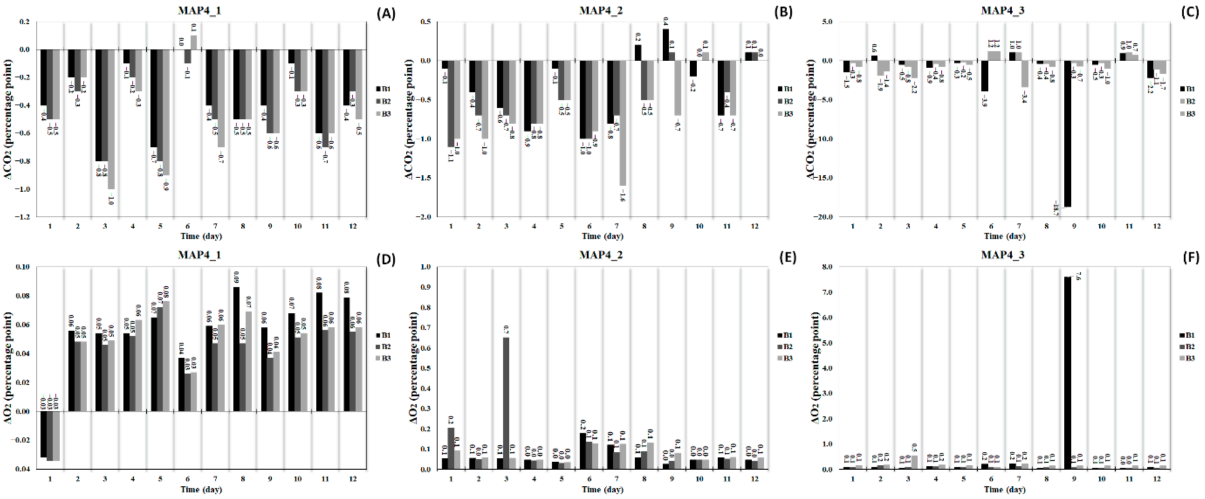

Figure S1. Overnight changes in headspace CO_2 and O_2 concentration in 1st bottle (B1), 2nd bottle (B2) and 3rd bottle (B3) under the following condition CO_2%/O_2%/N_2%: 60/0/40 at 7 °C for MAP4_1: 1st experiment (A and D), MAP4_2: 2nd experiment (B and E), and MAP4_3: 3rd experiment (C and F).

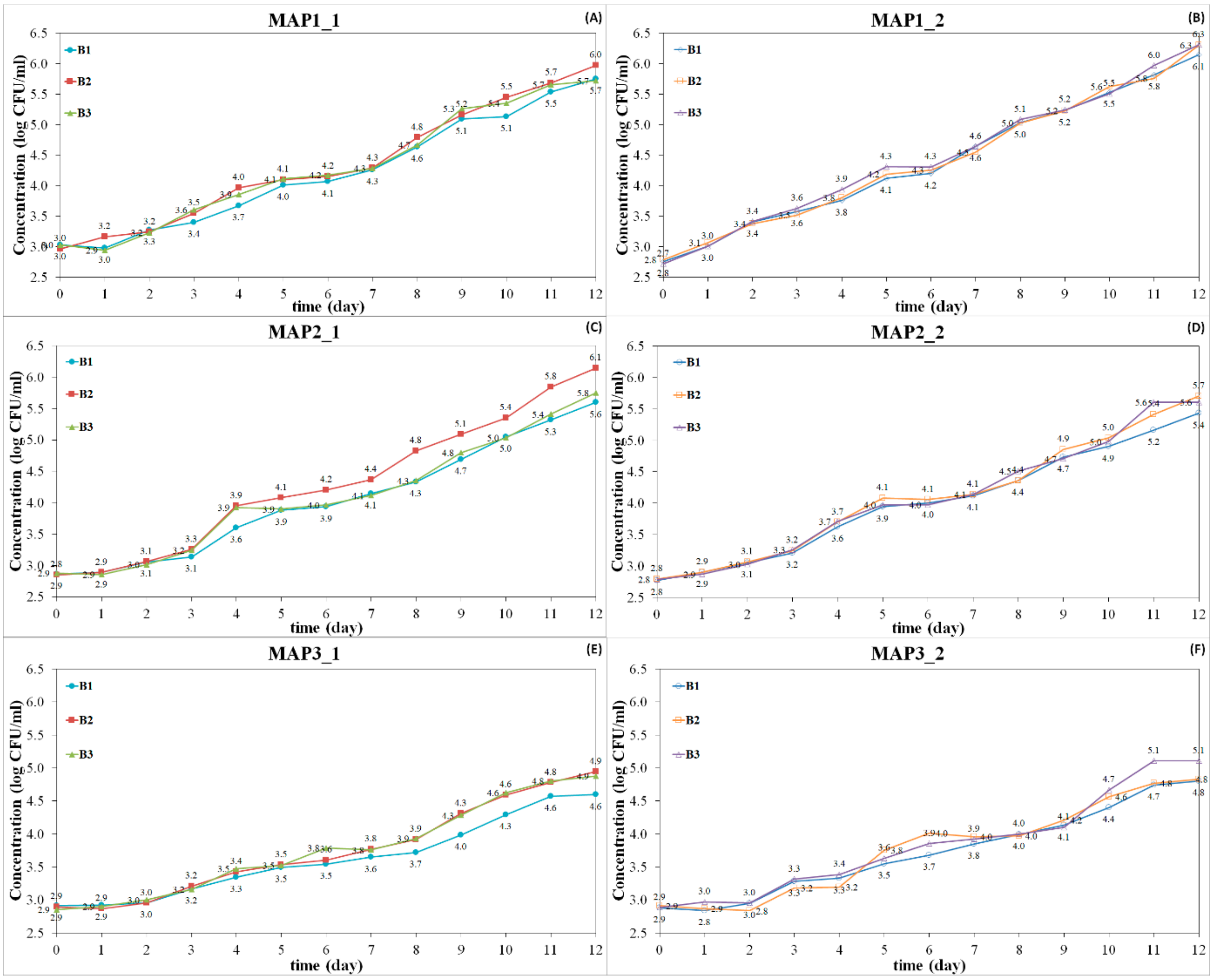

Figure S2. Growth curves obtained from 1st bottle (B1), 2nd bottle (B2) and 3rd bottle (B3) for following conditions CO_2%/O_2%/N_2%: 20/0/80 for MAP1_1: 1st experiment (A) and for MAP1_2: 2nd experiment (B), 40/0/60 for MAP2_1: 1st experiment (C) and MAP2_2: 2nd experiment (D), 60/0/40 for MAP3_1: 1st experiment (E) and MAP3_2: 2nd experiment (F).

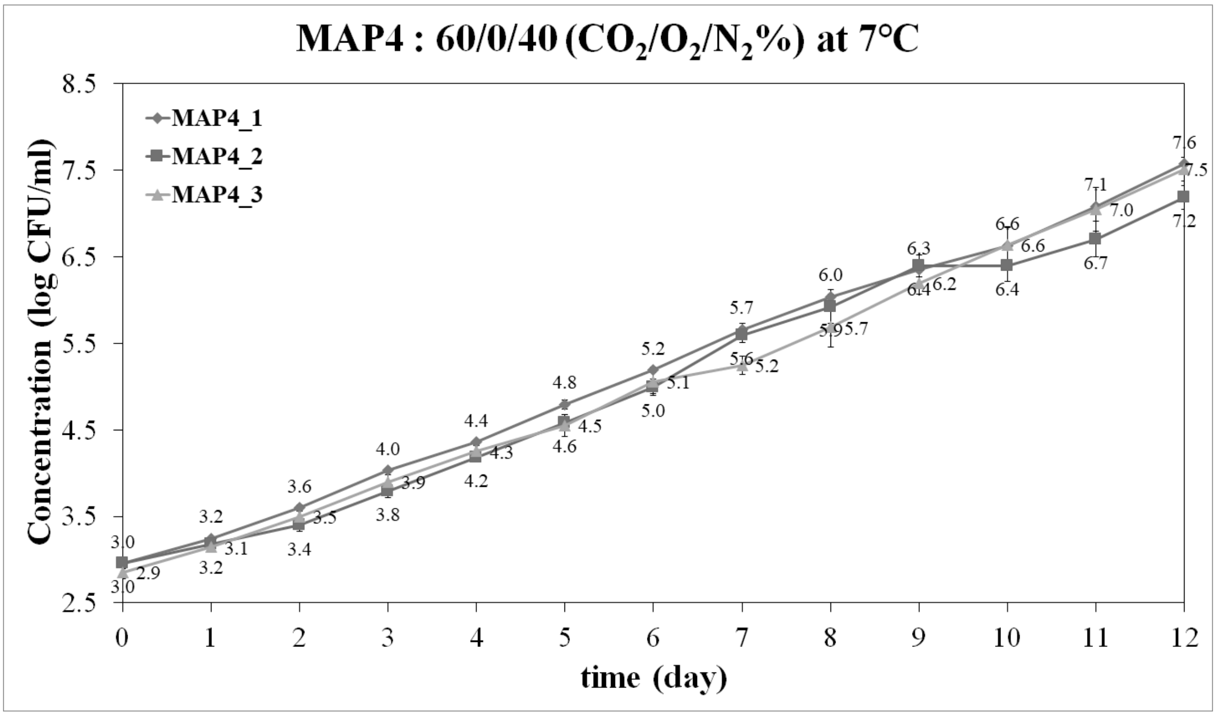

Figure S3. Growth curves of *L. monocytogenes* within a 13-day experiment under the following condition CO_2%/O_2%/N_2%: 60/0/40 for MAP4_1: 1st experiment, MAP4_2: 2nd experiment, and MAP4_3: 3rd experiment (error bars denote standard deviation, *n*=3 for MAP4_1 MAP4_2 and MAP4_3).
